# Supplementary material for: Evaluation of Interferon-Gamma Polymorphisms as a Risk factor in Feline Infectious Peritonitis Development in Non-Pedigree Cats—a Large Cohort Study
Source: Pathogens. 2020 Jul 3;9(7):535. doi: 10.3390/pathogens9070535 (PMC7399832; doi:10.3390/pathogens9070535)
Supplement: Supplementary file 1 [file pathogens-09-00535-s001.pdf]

Supplementary Data: Group, signalment data, diagnosis, and sequencing results for the cats included in the study. Group (FIP or General Population); cat identification number; age (in months) at time of FIP diagnosis, death, loss from follow-up, or at 1st April 2020 if alive; diagnosis (FIP; alive; lost to follow-up; died, and cause where recorded); feline interferon-gamma gene single nucleotide polymorphisms at g.401 and g.408; reason for exclusion (where applicable)]
?, unknown; DSH, domestic short-haired; DLH, domestic long-haired; n/a, not available

**Group**	**Reference**	**Age (months)**	**Breed**	**Sex**	**Diagnosis**	**g.401**	**g.408**	**Exclusions / Failure to sequence**
**FIP**	**FIP-UoB-001**	4	DSH	Male	FIP	C/T	C/T	
**FIP**	**FIP-UoB-002**	2	DSH	?	FIP	C/T	C/T	
**FIP**	**FIP-UoB-003**	4.5	DSH	?	FIP	C/T	C/T	
**FIP**	**FIP-UoB-004**	?	DSH	?	FIP	C/T	C/T	
**FIP**	**FIP-UoB-005**	?	DSH	?	FIP	C/T	C/T	
**FIP**	**FIP-UoB-006**	4	DSH	?	FIP	T/T	T/T	
**FIP**	**FIP-UoB-007**	?	DSH	?	FIP	C/T	C/T	
**FIP**	**FIP-UoB-008**	5	DSH	?	FIP	C/C	C/C	
**FIP**	**FIP-UoB-009**	4	DSH	?	FIP	C/C	C/C	
**FIP**	**FIP-UoB-010**	7	DSH	?	FIP	C/C	C/C	
**FIP**	**FIP-UoB-011**	22	DSH	Female	FIP	C/T	C/T	
**FIP**	**FIP-UoB-012**	?	DSH	Male	FIP	C/T	C/T	
**FIP**	**FIP-UoB-013**	9	DSH	Female	FIP	T/T	T/T	
**FIP**	**FIP-UoB-014**	?	DSH	?	FIP	C/T	C/T	
**FIP**	**FIP-UoB-015**	14	DSH	?	FIP	C/T	C/T	
**FIP**	**FIP-UoB-016**	?	DSH	?	FIP	T/T	T/T	
**FIP**	**FIP-UoB-017**	36	DSH	Male	FIP	C/T	C/T	
**FIP**	**FIP-UoB-018**	?	DSH	Male	FIP	T/T	T/T	
**FIP**	**FIP-UoB-019**	24	DSH	Male	FIP	C/T	C/T	
**FIP**	**FIP-UoB-020**	3	DSH	Male	FIP	C/C	C/C	
**FIP**	**FIP-UoB-021**	12	DSH	Male	FIP	C/T	C/T	
**FIP**	**FIP-UoB-022**	21	DSH	Male	FIP	C/C	C/C	
**FIP**	**FIP-UoB-023**	48	DSH	Male	FIP	C/C	C/C	

Group	Reference	Age (months)	Breed	Sex	Diagnosis	g.401	g.408	Exclusions / Failure to sequence
**FIP**	**FIP-UoB-024**	?	DSH	Male	FIP	C/C	C/C	
**FIP**	**FIP-UoB-025**	6	DSH	Female	FIP	C/T	C/T	
**FIP**	**FIP-UoB-026**	23	DSH	Male	FIP	C/T	C/T	
**FIP**	**FIP-UoB-027**	15	DSH	Male	FIP	C/T	C/T	
**FIP**	**FIP-UoB-028**	4.5	DSH	Female	FIP	C/T	C/T	
**FIP**	**FIP-UoB-029**	10	DSH	Male	FIP	C/T	C/T	
**FIP**	**FIP-UoB-030**	7	DSH	Male	FIP	C/T	C/T	
**FIP**	**FIP-UoB-031**	44	DSH	Male	FIP	C/C	C/C	
**FIP**	**FIP-UoB-032**	12	DSH	Female	FIP	C/T	C/T	
**FIP**	**FIP-UoB-033**	12	DSH	Female	FIP	T/T	T/T	
**FIP**	**FIP-UoB-034**	5.5	DSH	Female	FIP	C/C	C/C	
**FIP**	**FIP-UoL-001**	?	DSH	?	FIP	C/T	C/T	
**FIP**	**FIP-UoL-002**	56	DSH	Male	FIP	C/C	C/C	
**FIP**	**FIP-UoL-003**	12	DSH	Male	FIP	C/C	C/C	
**FIP**	**FIP-UoL-004**	10	DSH	Male	FIP	C/T	C/T	Duplicate
**FIP**	**FIP-UoL-005**	23	DSH	Male	FIP	C/T	C/T	
**FIP**	**FIP-UoL-006**	?	DSH	?	FIP	C/C	C/C	
**FIP**	**FIP-UoL-007**	10	DSH	Male	FIP	C/T	C/T	
**FIP**	**FIP-UoL-008**	3	DSH	Female	FIP	T/T	T/T	
**FIP**	**FIP-UoL-009**	12	DSH	Male	FIP	C/T	C/T	
**FIP**	**FIP-UoL-010**	6	DSH	Female	FIP	C/T	C/T	
**FIP**	**FIP-UoL-011**	?	DSH	?	FIP	T/T	T/T	
**FIP**	**FIP-UoL-012**	5	DSH	Male	FIP	C/T	C/T	
**FIP**	**FIP-UoL-013**	60	DSH	Male	FIP	T/T	T/T	
**FIP**	**FIP-UoL-014**	5	DSH	Male	FIP	C/C	C/C	
**FIP**	**FIP-UoL-015**	30	DSH	Male	FIP	C/T	C/T	

Group	Reference	Age (months)	Breed	Sex	Diagnosis	g.401	g.408	Exclusions / Failure to sequence
**FIP**	**FIP-UoL-016**	156	DSH	Male	FIP	C/T	C/T	
**FIP**	**FIP-UoL-017**	168	DSH	Male	FIP	C/T	C/T	
**FIP**	**FIP-UoL-018**	72	DSH	Male	FIP	C/T	C/T	
**FIP**	**FIP-UoL-019**	104	DSH	Male	FIP	C/C	C/C	
**FIP**	**FIP-UoL-020**	12	DSH	Female	FIP	C/C	C/C	
**FIP**	**FIP-UoL-021**	132	DLH	Male	FIP	T/T	T/T	
**FIP**	**FIP-UoL-022**	72	DSH	Male	FIP	C/T	C/T	
**FIP**	**FIP-UoL-023**	132	DSH	Male	FIP	C/C	C/C	
**FIP**	**FIP-UoL-024**	11	DSH	Female	FIP	N/A	N/A	Sequence failure
**FIP**	**FIP-UoL-025**	13	DSH	Male	FIP	N/A	N/A	Sequence failure

Group	Reference	Age (months)	Breed	Sex	Diagnosis	g401	g408	
**General Population**	**GP-BC-001**	92	DSH	Male	Alive	CT	CT	
**General Population**	**GP-BC-002**	109	DSH	Female	Alive	TT	TT	
**General Population**	**GP-BC-003**	93	DSH	Female	Alive	CC	CC	
**General Population**	**GP-BC-004**	80	DSH	Male	Alive	CC	CC	
**General Population**	**GP-BC-005**	91	DSH	Female	Alive	TT	TT	
**General Population**	**GP-BC-006**	107	DSH	Female	Alive	TT	TT	
**General Population**	**GP-BC-007**	91	DSH	Female	Alive	TT	TT	
**General Population**	**GP-BC-008**	81	DLH	Male	Alive	CC	CC	
**General Population**	**GP-BC-009**	98	DSH	Female	Alive	CT	CT	
**General Population**	**GP-BC-010**	78	DSH	Male	Alive	TT	TT	
**General Population**	**GP-BC-011**	96	DSH	Male	Alive	CT	CT	
**General Population**	**GP-BC-012**	118	DSH	Male	Alive	TT	TT	
**General Population**	**GP-BC-013**	91	DSH	Male	Alive	CT	CT	

Group	Reference	Age (months)	Breed	Sex	Diagnosis	g.401	g.408	Exclusions / Failure to sequence
**General Population**	**GP-BC-014**	91	DSH	Male	Alive	CC	CC	
**General Population**	**GP-BC-015**	118	DSH	Female	Alive	N/A	N/A	Sequence failure
**General Population**	**GP-BC-016**	105	DSH	Male	Alive	CT	CT	
**General Population**	**GP-BC-017**	84	DSH	Female	Alive	TT	TT	
**General Population**	**GP-BC-018**	107	DSH	Male	Alive	CT	CT	
**General Population**	**GP-BC-019**	115	DSH	male	Alive	CC	CC	
**General Population**	**GP-BC-020**	97	DSH	Male	Alive	CC	CC	
**General Population**	**GP-BC-021**	85	DLH	Female	Alive	TT	TT	
**General Population**	**GP-BC-022**	15	DSH	Female	Died - RTA	CT	CT	
**General Population**	**GP-BC-023**	106	DSH	Male	Alive	CT	CT	
**General Population**	**GP-BC-024**	105	DSH	Female	Alive	CC	CC	
**General Population**	**GP-BC-025**	108	DSH	Male	Alive	TT	TT	
**General Population**	**GP-BC-026**	102	DLH	Female	Alive	TT	TT	
**General Population**	**GP-BC-027**	102	DSH	Female	Alive	TT	TT	
**General Population**	**GP-BC-028**	107	DSH	Female	Alive	TT	TT	
**General Population**	**GP-BC-029**	105	DSH	Female	Alive	CT	CT	
**General Population**	**GP-BC-030**	104	DSH	Female	Alive	TT	TT	
**General Population**	**GP-BC-031**	16	DSH	Female	Died - 'viral infection'	CC	CC	
**General Population**	**GP-BC-032**	104	DSH	Female	Alive	CC	CC	
**General Population**	**GP-BC-033**	91	DSH	Male	Alive	TT	TT	
**General Population**	**GP-BC-034**	105	DSH	Female	Alive	TT	TT	
**General Population**	**GP-BC-035**	105	DSH	Female	Alive	CT	CT	
**General Population**	**GP-BC-036**	105	DSH	Female	Alive	CT	CT	
**General Population**	**GP-BC-037**	104	DSH	Female	Alive	CT	CT	
**General Population**	**GP-BC-038**	104	DSH	Male	Alive	CT	CT	

Group	Reference	Age (months)	Breed	Sex	Diagnosis	g.401	g.408	Exclusions / Failure to sequence
**General Population**	**GP-BC-039**	110	DSH	Male	Alive	CC	CC	
**General Population**	**GP-BC-040**	49	DSH	Male	Died - neurological problems	CT	CT	
**General Population**	**GP-BC-041**	89	DSH	Male	Alive	TT	TT	
**General Population**	**GP-BC-042**	7	DSH	Female	Died - anesthetic complications at neutering	CC	CC	
**General Population**	**GP-BC-043**	104	DSH	Female	Alive	CT	CT	
**General Population**	**GP-BC-044**	109	DSH	Male	Alive	CT	CT	
**General Population**	**GP-BC-045**	91	DSH	Male	Alive	CT	CT	
**General Population**	**GP-BC-046**	49	DSH	Male	Died - RTA	TT	TT	
**General Population**	**GP-BC-047**	104	DSH	Male	Alive	CC	CC	
**General Population**	**GP-BC-048**	104	DSH	Male	Alive	CT	CT	
**General Population**	**GP-BC-049**	104	DSH	Female	Alive	CT	CT	
**General Population**	**GP-BC-050**	109	DSH	Female	Alive	CT	CT	
**General Population**	**GP-BC-051**	105	DSH	Male	Alive	CT	CT	
**General Population**	**GP-BC-052**	80	DSH	Female	Alive	CT	CT	
**General Population**	**GP-BC-053**	109	DSH	Female	Alive	CT	CT	
**General Population**	**GP-BC-054**	109	DSH	Male	Alive	CT	CT	
**General Population**	**GP-BC-055**	120	DSH	Male	Alive	CC	CC	
**General Population**	**GP-BC-056**	105	DLH	Male	Alive	TT	TT	
**General Population**	**GP-BC-057**	68	DSH	Female	Died - *Toxoplasma* meningoencephalitis	CT	CT	
**General Population**	**GP-BC-058**	95	DSH	Male	Alive	CC	CC	
**General Population**	**GP-BC-059**	84	DSH	Male	Alive	CT	CT	
**General Population**	**GP-BC-060**	81	DSH	Female	Alive	CT	CT	
**General Population**	**GP-BC-061**	84	DSH	Female	Lost to follow-up	TT	TT	
**General Population**	**GP-BC-062**	87	DLH	Female	Alive	TT	TT	
**General Population**	**GP-BC-063**	93	DLH	Male	Alive	CT	CT	

Group	Reference	Age (months)	Breed	Sex	Diagnosis	g.401	g.408	Exclusions / Failure to sequence
**General Population**	**GP-BC-064**	107	DSH	female	Alive	TT	TT	
**General Population**	**GP-BC-065**	92	DLH	Male	Alive	CC	CC	
**General Population**	**GP-BC-066**	116	DSH	male	Alive	CC	CC	
**General Population**	**GP-BC-067**	106	DSH	Male	Alive	CC	CC	
**General Population**	**GP-BC-068**	82	DLH	Male	Alive	TT	TT	
**General Population**	**GP-BC-069**	78	DSH	Female	Alive	CT	CT	
**General Population**	**GP-BC-070**	83	DSH	Female	Alive	CT	CT	
**General Population**	**GP-BC-071**	85	DSH	Male	Alive	CC	CC	
**General Population**	**GP-BC-072**	95	DSH	Female	Alive	TT	TT	
**General Population**	**GP-BC-073**	83	DSH	Male	Alive	TT	TT	
**General Population**	**GP-BC-074**	83	DSH	Male	Alive	CC	CC	
**General Population**	**GP-BC-075**	92	DSH	Female	Alive	TT	TT	
**General Population**	**GP-BC-076**	85	DSH	Male	Alive	CT	CT	
**General Population**	**GP-BC-077**	96	DLH	Female	Alive	CC	CC	
**General Population**	**GP-BC-078**	75	DSH	Female	Lost to follow-up	CT	CT	
**General Population**	**GP-BC-079**	86	DSH	Male	Alive	CT	CT	
**General Population**	**GP-BC-080**	95	DSH	Female	Alive	TT	TT	
**General Population**	**GP-BC-081**	48	DSH	Male	Died - RTA	CT	CT	
**General Population**	**GP-BC-082**	83	DSH	Male	Alive	CT	CT	
**General Population**	**GP-BC-083**	62	DSH	Male	Died - heart disease	CT	CT	
**General Population**	**GP-BC-084**	90	DSH	Male	Alive	CC	CC	
**General Population**	**GP-BC-085**	78	DSH	Male	Alive	CT	CT	
**General Population**	**GP-BC-086**	93	DLH	Male	Alive	CT	CT	
**General Population**	**GP-BC-087**	83	DSH	Male	Alive	TT	TT	
**General Population**	**GP-BC-088**	90	DSH	Male	Alive	TT	TT	

Group	Reference	Age (months)	Breed	Sex	Diagnosis	g.401	g.408	Exclusions / Failure to sequence
**General Population**	**GP-BC-089**	93	DSH	Female	Alive	TT	TT	
**General Population**	**GP-BC-090**	92	DSH	Female	Alive	CT	CT	
**General Population**	**GP-BC-091**	61	DSH	Male	Died - RTA	CC	CC	
**General Population**	**GP-BC-092**	92	DSH	Male	Alive	CT	CT	
**General Population**	**GP-BC-093**	84	DSH	Male	Alive	CT	CT	
**General Population**	**GP-BC-094**	86	DSH	Male	Alive	CT	CT	
**General Population**	**GP-BC-095**	74	DSH	male	Died - heart disease	CT	CT	
**General Population**	**GP-BC-096**	107	DSH	Male	Alive	TT	TT	
**General Population**	**GP-BC-097**	120	DSH	Female	Alive	CT	CT	
**General Population**	**GP-BC-098**	51	DSH	Female	Died - unknown cause	TT	TT	
**General Population**	**GP-BC-099**	86	DSH	Male	Lost to follow-up	TT	TT	
**General Population**	**GP-BC-100**	118	DSH	Female	Alive	TT	TT	
**General Population**	**GP-BC-101**	86	DSH	Female	Died - trauma	TT	TT	
**General Population**	**GP-BC-102**	106	DLH	Male	Alive	CC	CC	
**General Population**	**GP-BC-103**	95	DSH	male	Died - kidney disease	TT	TT	
**General Population**	**GP-BC-104**	106	DSH	Female	Alive	CT	CT	
**General Population**	**GP-BC-105**	109	DSH	Male	Alive	TT	TT	
**General Population**	**GP-BC-106**	109	DSH	Male	Alive	CT	CT	
**General Population**	**GP-BC-107**	110	DSH	Male	Alive	CC	CC	
**General Population**	**GP-BC-108**	106	DLH	Female	Alive	TT	TT	
**General Population**	**GP-BC-109**	120	DSH	Male	Alive	CC	CC	
**General Population**	**GP-BC-110**	117	DLH	Male	Alive	CC	CC	
**General Population**	**GP-BC-111**	120	DSH	Female	Alive	CT	CT	
**General Population**	**GP-BC-112**	9	DSH	Female	Died - RTA	TT	TT	
**General Population**	**GP-BC-113**	120	DSH	Female	Alive	CT	CT	

Group	Reference	Age (months)	Breed	Sex	Diagnosis	g.401	g.408	Exclusions / Failure to sequence
**General Population**	**GP-BC-114**	108	DSH	Male	Alive	CC	CC	
**General Population**	**GP-BC-115**	105	DSH	Male	Lost to follow-up	CT	CT	
**General Population**	**GP-BC-116**	109	DSH	Male	Alive	CT	CT	
**General Population**	**GP-BC-117**	109	DSH	Male	Alive	CT	CT	
**General Population**	**GP-BC-118**	109	DLH	Female	Alive	CC	CC	
**General Population**	**GP-BC-119**	101	DSH	female	Died - RTA	TT	TT	
**General Population**	**GP-BC-120**	118	DSH	Female	Alive	CT	CT	
**General Population**	**GP-BC-121**	52	DSH	Male	Lost to follow-up	TT	TT	
**General Population**	**GP-BC-122**	119	DLH	Male	Alive	TT	TT	
**General Population**	**GP-BC-123**	106	DSH	Female	Alive	TT	TT	
**General Population**	**GP-BC-124**	117	DSH	Male	Alive	CC	CC	
**General Population**	**GP-BC-125**	85	DSH	Female	Died - unknown cause	CC	CC	
**General Population**	**GP-BC-126**	108	DSH	Male	Alive	TT	TT	
**General Population**	**GP-BC-127**	119	DSH	Male	Alive	CC	CC	
**General Population**	**GP-BC-128**	69	DSH	Male	Died - RTA	CT	CT	
**General Population**	**GP-BC-129**	116	DSH	female	Alive	TT	TT	
**General Population**	**GP-BC-130**	72	DSH	male	Died - unknown cause	TT	TT	
**General Population**	**GP-BC-131**	109	DSH	Male	Alive	CT	CT	
**General Population**	**GP-BC-132**	108	DSH	Male	Alive	CC	CC	
**General Population**	**GP-BC-133**	36	DSH	Male	Died - kidney disease	CT	CT	
**General Population**	**GP-BC-134**	108	DSH	Female	Alive	CC	CC	
**General Population**	**GP-BC-135**	32	DSH	Male	Died - RTA	CT	CT	
**General Population**	**GP-BC-136**	107	DSH	Female	Alive	CC	CC	
**General Population**	**GP-BC-137**	19	DSH	Male	Died - RTA	CC	CC	
**General Population**	**GP-BC-138**	119	DSH	Female	Alive	TT	TT	

Group	Reference	Age (months)	Breed	Sex	Diagnosis	g.401	g.408	Exclusions / Failure to sequence
**General Population**	**GP-BC-139**	107	DSH	Female	Alive	CT	CT	
**General Population**	**GP-BC-140**	63	DSH	Male	Died - kidney disease	TT	TT	
**General Population**	**GP-BC-141**	108	DSH	Male	Alive	CT	CT	
**General Population**	**GP-BC-142**	9	DSH	Female	Died - RTA	TT	TT	
**General Population**	**GP-BC-143**	61	DSH	Female	Died - RTA	CT	CT	
**General Population**	**GP-BC-144**	106	DSH	Male	Alive	CC	CC	
**General Population**	**GP-BC-145**	107	DSH	Female	Alive	TT	TT	
**General Population**	**GP-BC-146**	18	DSH	Male	Died - RTA	TT	TT	
**General Population**	**GP-BC-147**	108	DSH	Female	Alive	TT	TT	
**General Population**	**GP-BC-148**	119	DLH	Female	Alive	CC	CC	
**General Population**	**GP-BC-149**	109	DLH	Female	Alive	CT	CT	
**General Population**	**GP-BC-150**	109	DLH	Female	Alive	CC	CC	
**General Population**	**GP-BC-151**	108	DSH	Female	Alive	TT	TT	
**General Population**	**GP-BC-152**	54	DLH	Male	Died - unknown cause	TT	TT	
**General Population**	**GP-BC-153**	107	DLH	Male	Alive	CT	CT	
**General Population**	**GP-BC-154**	119	DSH	Female	Alive	CC	CC	
**General Population**	**GP-BC-155**	32	DSH	Female	Died - unknown cause	TT	TT	
**General Population**	**GP-BC-156**	9	DLH	Male	Died - RTA	CT	CT	
**General Population**	**GP-BC-157**	109	DSH	Male	Alive	TT	TT	
**General Population**	**GP-BC-158**	109	DSH	Female	Alive	CT	CT	
**General Population**	**GP-BC-159**	109	DSH	Female	Alive	CT	CT	
**General Population**	**GP-BC-160**	108	DSH	Female	Alive	TT	TT	
**General Population**	**GP-BC-161**	101	DSH	Male	Died - cancer	CT	CT	
**General Population**	**GP-BC-162**	109	DSH	Male	Alive	CT	CT	
**General Population**	**GP-BC-163**	96	DSH	Male	Lost to follow-up	TT	TT	

Group	Reference	Age (months)	Breed	Sex	Diagnosis	g.401	g.408	Exclusions / Failure to sequence
**General Population**	**GP-BC-164**	118	DSH	Male	Alive	TT	TT	
**General Population**	**GP-BC-165**	109	DSH	Female	Alive	CC	CC	
**General Population**	**GP-BC-166**	106	DSH	Male	Alive	TT	TT	
**General Population**	**GP-BC-167**	118	DSH	Female	Alive	CT	CT	
**General Population**	**GP-BC-168**	107	DSH	Female	Alive	TT	TT	
**General Population**	**GP-BC-169**	85	DSH	Female	Died - kidney disease	TT	TT	
**General Population**	**GP-BC-170**	109	DSH	male	Lost to follow-up	CC	CC	
**General Population**	**GP-BC-171**	109	DLH	Male	Alive	CT	CT	
**General Population**	**GP-BC-172**	108	DLH	Male	Alive	TT	TT	
**General Population**	**GP-BC-173**	108	DSH	Female	Alive	TT	TT	
**General Population**	**GP-BC-174**	62	DSH	Female	Died - kidney disease	CC	CC	
**General Population**	**GP-BC-175**	28	DSH	Female	Died - RTA	TT	TT	
**General Population**	**GP-BC-176**	118	DSH	Male	Alive	TT	TT	
**General Population**	**GP-BC-177**	117	DSH	Male	Alive	CT	CT	
**General Population**	**GP-BC-178**	103	DSH	Male	Alive	TT	TT	
**General Population**	**GP-BC-179**	26	DSH	Male	Died - behavioural issues	CC	CC	
**General Population**	**GP-BC-180**	107	DSH	Male	Alive	TT	TT	
**General Population**	**GP-BC-181**	103	DSH	Female	Alive	TT	TT	
**General Population**	**GP-BC-182**	103	DLH	Female	Alive	CT	CT	
**General Population**	**GP-BC-183**	107	DLH	Male	Alive	TT	TT	
**General Population**	**GP-BC-184**	39	DSH	Male	Died - trauma	CC	CC	
**General Population**	**GP-BC-185**	103	DSH	Female	Alive	CT	CT	
**General Population**	**GP-BC-186**	10	DSH	Female	Died - feline dysautonomia	CT	CT	
**General Population**	**GP-BC-187**	94	DSH	Female	Lost to follow-up	CC	CC	
**General Population**	**GP-BC-188**	14	DSH	Male	Died - RTA	CT	CT	

Group	Reference	Age (months)	Breed	Sex	Diagnosis	g.401	g.408	Exclusions / Failure to sequence
**General Population**	**GP-BC-189**	104	DSH	Male	Alive	TT	TT	
**General Population**	**GP-BC-190**	107	DSH	Male	Alive	CT	CT	
**General Population**	**GP-BC-191**	106	DSH	Male	Alive	CT	CT	
**General Population**	**GP-BC-192**	104	DSH	Female	Alive	CT	CT	
**General Population**	**GP-BC-193**	9	DSH	Male	Died - RTA	CT	CT	
**General Population**	**GP-BC-194**	16	DSH	Female	Died - RTA	CT	CT	
**General Population**	**GP-BC-195**	107	DSH	Female	Alive	CT	CT	
**General Population**	**GP-BC-196**	107	DSH	Female	Alive	TT	TT	
**General Population**	**GP-BC-197**	106	DSH	Male	Alive	CT	CT	
**General Population**	**GP-BC-198**	101	DLH	Male	Alive	TT	TT	
**General Population**	**GP-BC-199**	7	DLH	Female	Died - RTA	CC	CC	
**General Population**	**GP-BC-200**	107	DLH	Male	Alive	TT	TT	
**General Population**	**GP-BC-201**	105	DSH	Male	Alive	CT	CT	
**General Population**	**GP-BC-202**	79	DSH	Female	Died - neurological disease	CT	CT	
**General Population**	**GP-BC-203**	104	DSH	Male	Alive	CT	CT	
**General Population**	**GP-BC-204**	105	DLH	Male	Alive	TT	TT	
**General Population**	**GP-BC-205**	103	DSH	Male	Alive	TT	TT	
**General Population**	**GP-BC-206**	105	DLH	Female	Alive	CT	CT	
**General Population**	**GP-BC-207**	105	DSH	Male	Alive	TT	TT	
**General Population**	**GP-BC-208**	102	DSH	Male	Alive	CT	CT	
**General Population**	**GP-BC-209**	74	DSH	Male	Died - cancer	CT	CT	
**General Population**	**GP-BC-210**	106	DSH	Male	Alive	TT	TT	
**General Population**	**GP-BC-211**	107	DSH	Male	Alive	CT	CT	
**General Population**	**GP-BC-212**	105	DLH	Male	Alive	TT	TT	
**General Population**	**GP-BC-213**	31	DLH	Male	Died - pyothorax	TT	TT	

Group	Reference	Age (months)	Breed	Sex	Diagnosis	g.401	g.408	Exclusions / Failure to sequence
**General Population**	**GP-BC-214**	108	DSH	Female	Alive	TT	TT	
**General Population**	**GP-BC-215**	103	DSH	Male	Alive	CC	CC	
**General Population**	**GP-BC-216**	10	DSH	Female	Died - RTA	CT	CT	
**General Population**	**GP-BC-217**	100	DSH	Male	Alive	CC	CC	
**General Population**	**GP-BC-218**	97	DSH	Male	Alive	TT	TT	
**General Population**	**GP-BC-219**	97	DSH	Female	Alive	CT	CT	
**General Population**	**GP-BC-220**	97	DSH	Male	Alive	TT	TT	
**General Population**	**GP-BC-221**	97	DLH	Male	Alive	CT	CT	
**General Population**	**GP-BC-222**	101	DSH	Female	Alive	CT	CT	
**General Population**	**GP-BC-223**	21	DSH	Female	Died - RTA	CT	CT	
**General Population**	**GP-BC-224**	97	DSH	Female	Alive	CT	CT	
**General Population**	**GP-BC-225**	14	DSH	Male	Died - RTA	CT	CT	
**General Population**	**GP-BC-226**	97	DSH	Male	Alive	CT	CT	
**General Population**	**GP-BC-227**	102	DSH	Female	Alive	CT	CT	
**General Population**	**GP-BC-228**	101	DSH	Female	Alive	TT	TT	
**General Population**	**GP-BC-229**	102	DSH	Male	Alive	CT	CT	
**General Population**	**GP-BC-230**	99	DLH	Male	Alive	TT	TT	
**General Population**	**GP-BC-231**	94	DLH	male	Died - unknown cause	CT	CT	
**General Population**	**GP-BC-232**	101	DLH	male	Alive	CT	CT	
**General Population**	**GP-BC-233**	96	DSH	Female	Alive	CT	CT	
**General Population**	**GP-BC-234**	19	DSH	Male	Died - RTA	CT	CT	
**General Population**	**GP-BC-235**	97	DSH	Female	Alive	TT	TT	
**General Population**	**GP-BC-236**	118	DSH	Male	Alive	CC	CC	
**General Population**	**GP-BC-237**	24	DSH	Male	Died - RTA	CC	CC	
**General Population**	**GP-BC-238**	97	DSH	Female	Alive	CC	CC	

Group	Reference	Age (months)	Breed	Sex	Diagnosis	g.401	g.408	Exclusions / Failure to sequence
**General Population**	**GP-BC-239**	84	DSH	Male	Lost to follow-up	CC	CC	
**General Population**	**GP-BC-240**	85	DSH	Male	Died - cancer	CC	CC	
**General Population**	**GP-BC-241**	79	DSH	Female	Alive	CT	CT	
**General Population**	**GP-BC-242**	79	DSH	Female	Alive	TT	TT	
**General Population**	**GP-BC-243**	93	DSH	Female	Alive	CT	CT	
**General Population**	**GP-BC-244**	83	DSH	Female	Alive	CT	CT	
**General Population**	**GP-BC-245**	95	DSH	Male	Alive	CT	CT	
**General Population**	**GP-BC-246**	81	DSH	Male	Alive	CC	CC	
**General Population**	**GP-BC-247**	92	DSH	Female	Alive	CT	CT	
**General Population**	**GP-BC-248**	92	DSH	Male	Alive	CT	CT	
**General Population**	**GP-BC-249**	92	DSH	Male	Alive	CT	CT	
**General Population**	**GP-BC-250**	84	DSH	Female	Alive	CT	CT	
**General Population**	**GP-BC-251**	80	DSH	Female	Alive	CT	CT	
**General Population**	**GP-BC-252**	83	DSH	Male	Alive	CT	CT	
**General Population**	**GP-BC-253**	91	DLH	Female	Alive	CT	CT	
**General Population**	**GP-BC-254**	96	DSH	Male	Alive	CC	CC	
**General Population**	**GP-BC-255**	80	DSH	Male	Alive	CT	CT	
**General Population**	**GP-BC-256**	80	DSH	Male	Alive	CT	CT	
**General Population**	**GP-BC-257**	84	DSH	Male	Alive	CT	CT	
**General Population**	**GP-BC-258**	93	DLH	Male	Alive	TT	TT	
**General Population**	**GP-BC-259**	80	DSH	Female	Alive	CC	CC	
**General Population**	**GP-BC-260**	92	DSH	Female	Alive	TT	TT	
**General Population**	**GP-BC-261**	95	DSH	Male	Alive	CT	CT	
**General Population**	**GP-BC-262**	85	DSH	Female	Died - RTA	TT	TT	
**General Population**	**GP-BC-263**	109	DSH	Male	Alive	TT	TT	

Group	Reference	Age (months)	Breed	Sex	Diagnosis	g.401	g.408	Exclusions / Failure to sequence
**General Population**	**GP-BC-264**	107	DSH	Female	Alive	CC	CC	
